# Renal Resistive Index: Revisited

**DOI:** 10.7759/cureus.36091

**Published:** 2023-03-13

**Authors:** Theertha K.C., Sudha K Das, Manjunath S Shetty

**Affiliations:** 1 Department of Radiology, JSS Medical College and Hospital, JSS Academy of Higher Education and Research, Mysuru, IND; 2 Department of Nephrology, JSS Medical College and Hospital, JSS Academy of Higher Education and Research, Mysuru, IND

**Keywords:** diabetic nephropathy, egfr, renal resistive index, duplex doppler sonography, chronic kidney disease

## Abstract

Introduction

Chronic kidney disease (CKD) is universally considered a public health burden and the majority of cases are found to be diabetic at the time of diagnosis. Renal biopsy is the prime modality for the complete evaluation of renal injuries but is invasive. Duplex Doppler sonography can help to determine renal resistive index (RRI), which is an excellent marker for demonstrating dynamic or structural changes of intrarenal vessels. In this study, we evaluated the intrarenal hemodynamic abnormalities with RRI in diabetic and non-diabetic kidney disease patients. Also, RRI was correlated with the established parameters of renal dysfunction, i.e., estimated glomerular filtration rate (eGFR) and other biochemical parameters.

Results

There was a significant correlation of RRI with eGFR and serum creatinine indicating its role as a Doppler parameter, which can be used as complementary to biochemical parameters. A remarkable difference was noted in the RRI values between diabetic and non-diabetic groups in the early stages of CKD, revealing its ability to arrive at etiopathogenesis in the early stages. The renal resistive index increases in a sequential pattern and is an indicator of declining renal function.

Conclusions

The addition of sonographic parameters like renal resistive index could help in the complete evaluation of chronic kidney disease in diabetic and non-diabetic groups. A sequential increase in renal resistive index is a better indicator of the progressive worsening of renal function as opposed to an absolute cut-off value.

## Introduction

Chronic kidney disease (CKD) is a worldwide health problem, and its prevalence and incidence are increasing. It is defined as renal injury for more than three months with or without a reduction in glomerular filtration rate (GFR), or a decrease in GFR for more than three months with or without kidney damage [1]. There are several risk factors that beget the development of CKD such as diabetes, hypertension, obesity, tobacco smoking, and dyslipidemia. Diabetes mellitus is the leading cause of CKD, and diabetic nephropathy (DN) accounts for 50% of prevalent kidney failure [2]. Early detection of diabetes and its microvascular complications is essential to reduce its burden. Renal biopsy is the gold standard modality for the evaluation of intrarenal damage. However, it has several drawbacks, such as high cost, invasiveness, sampling error, and low repeatability. Therefore, there is a critical need to develop a non-invasive, cheaper, and faster reproducible alternative to detect intrarenal damage.

Duplex Doppler sonography, which simultaneously measures both real-time and pulsed Doppler sonography, has been in clinical use since ages for sonographic detection of renal vessels [3]. The two main parameters used to describe intrarenal vascular resistance are the Pulsatility Index (PI) and the Resistive Index (RI). RI is the indicator of resistance of an organ to perfusion and reflects downstream resistance in arteries [4]. The normal scale for Renal Resistive Index ranges between 0.47-0.70, increases with age, and shows a difference of less than 5-8% between the two kidneys. Normal PI ranges between 0.7- 1.4 [5]. Elevated Renal Resistive Index (RRI) is markedly associated with renal arteriosclerosis (as a result of the scarring process) and adverse cardiovascular events [6].

Hence, the evaluation of intrarenal hemodynamic abnormalities using Doppler sonography could be an alternative to renal biopsy in detecting intrarenal damage. Also, this study may help identify CKD patients in their early stages and prevent them from going into end-stage renal disease.

This article was previously presented as an oral paper at the 75th annual conference of the Tamil Nadu and Pondicherry chapter of the Indian Radiological and Imaging Association (IRIA) on 18th December 2022.

## Materials and methods

This was a cross-sectional comparative study conducted on 114 patients (58 diabetic and 56 non-diabetic patients) over a period of 18 months (1st Dec 2020 to 31st May 2022) at the Department of Radiology, JSS Hospital, Mysuru. Patients were selected according to inclusion criteria, which included all CKD patients (with and without diabetes) who had undergone histopathological examination and were referred to the radiology department for ultrasound evaluation of the abdomen. The subjects excluded from the study were - aged less than 18 years, patients with renal artery stenosis/urinary tract obstruction, renal transplant recipients, and those undergoing renal replacement therapy.

After obtaining relevant clinical history and consent from the patients, they were subjected to routine ultrasound examination for renal volume calculation, followed by renal Doppler interrogation of the interlobar arteries using a 1-5 MHz convex probe.

Evaluation of the interlobar arteries in bilateral kidneys was done in a supine patient using colour Doppler. The sampling gate of Pulsed Doppler mode was placed at the mid portion of the interlobar arteries (2-4 mm of Doppler sample volume and <60 degrees of angle of insonation) at three poles (upper, middle, and lower) in each kidney. As a result, velocities and resistance parameters were obtained. The Renal Resistive Index was calculated as the mean of the six readings from both kidneys.

Patients' medical records were examined for various parameters like age, systolic blood pressure, urine albumin excretion, and serum creatinine, and used for the calculation of estimated GFR (eGFR). The patients were stratified into various stages of CKD based on eGFR. The trend of the Renal Resistive Index in varied stages of CKD was evaluated, with a further correlation of the Renal Resistive Index with other parameters such as age, urine albumin excretion, serum creatinine, and systolic blood pressure.

Statistical analysis

The lab values of renal function test (RFT) were obtained and eGFR was calculated by the CKD-EPI (CKD Epidemiology Collaboration) formula:

GFR (mL/min) = 141 x min(S Cr/K, 1)α x max(S Cr/K, 1)-1.209 x 0.993 Age x 1.018(if female) x 1.159(if black)

Where, K = 0.7 if female, 0.9 if male; α = -0.329 if female, -0.411 if male; min = the minimum of S Cr/K or 1; max = the maximum of S Cr/K or 1.

The renal resistive index was measured by duplex Doppler.

Analysis was performed using SPSS version 22 (IBM Corp., Armonk, USA) after entering the data into Microsoft Excel (Microsoft Corporation, Redmond, USA). Demographic characteristics and clinical parameters such as age, systolic blood pressure, serum creatinine, and urine albumin were represented using mean, standard deviation, and percentages. The comparison of RRI values between diabetic and non-diabetic kidney disease patients was done using the Independent sample t-test. The comparison of RRI values within diabetic and non-diabetic kidney disease patients across various stages of CKD was done using Analysis of Variance (ANOVA). The correlation of different categories of age, urine albumin, serum creatinine, systolic blood pressure, and eGFR with RRI was done using ANOVA. The commencement of hemodialysis was compared between the diabetic and non-diabetic groups of patients using ANOVA. Receiver operating characteristic (ROC) analysis was performed to determine a cut-off of RRI value to distinguish stage IV and above stages of CKD from the lower stages.

## Results

In our study, there was a slight male preponderance, with 62% of them being males and 38% being females. On dividing the patients into four age groups - 18-30 years (group 1), 31-45 years (group 2), 46-60 years (group 3), and above 60 years (group 4) - the majority of them were in the age group of 31-45 years (39%). Diabetic patients ranged from ages 24 to 73 years and non-diabetic patients ranged from 18 to 67 years. The mean age for diabetic patients (51 years) was significantly higher compared to non-diabetic patients (35 years) with a p-value of 0.001.

Stratification of patients into various stages of CKD showed that diabetic patients were significantly higher in the advanced stages compared to non-diabetic patients (p=0.007).

Subjects with diabetes showed significantly higher RRI values (mean - 0.72) than those without diabetes (mean - 0.65) (p=0.001). However, renal volume did not show any association with the presence/absence of diabetes (p=0.105).

**Figure 1:**
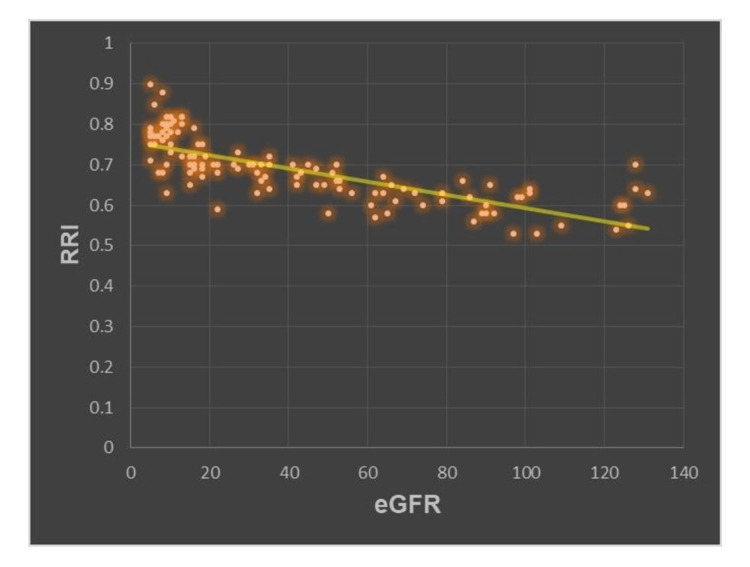
Scatter diagram illustrating the correlation of RRI and eGFR RRI: renal resistive index; eGFR: estimated glomerular filtration rate

The clinical and lab parameters (age, serum creatinine, urine albumin, systolic blood pressure, and eGFR) were divided into various categories and were correlated with RRI. The patients were classified into three groups based on RRI - normal RRI group (RRI <0.65), borderline RRI group (0.65 ≤ RRI < 0.7), and high RRI group (RRI ≥0.7). RRI was seen to be significantly associated with serum creatinine (p=0.001) and eGFR (p=0.001), however, it was not associated with patient age, systolic blood pressure, and urine albumin excretion. There was a remarkable negative correlation between RRI and eGFR in CKD patients, signifying that RRI progressively increased from the lower to the higher stages of CKD (Figure 1).

On the stage-wise distribution of mean RRI, no significant difference was noted in the mean RRI value between diabetic and non-diabetic kidney disease patients in stages I, IV, and V of CKD. However, a significant difference was noted in stages II and III (stage II: p= 0.002; stage III: p= 0.024) (Table 1).

**Table 1:** Comparison of the mean RRI based on diabetes and stages of CKD *significant; RRI: renal resistive index; CKD: chronic kidney disease; DM: diabetes mellitus

STAGE	DM	N	Minimum	Maximum	Mean	SD	Mean diff	p-value
I	Absent	13	.53	.70	.6008	.0550	-0.009	0.97
	Present	6	.58	.63	.6017	.0204		
II	Absent	12	.56	.66	.6033	.0290	-0.446	0.002*
	Present	10	.60	.70	.6480	.0305		
III	Absent	15	.58	.70	.6613	.0304	-0.031	0.024*
	Present	7	.65	.72	.6929	.0221		
IV	Absent	8	.65	.75	.7013	.0348	-0.0150	0.49
	Present	11	.68	.79	.7118	.0309		
V	Absent	8	.68	.82	.7550	.0504	-0.032	0.122
	Present	24	.70	.90	.7871	.0490		

**Figure 2:**
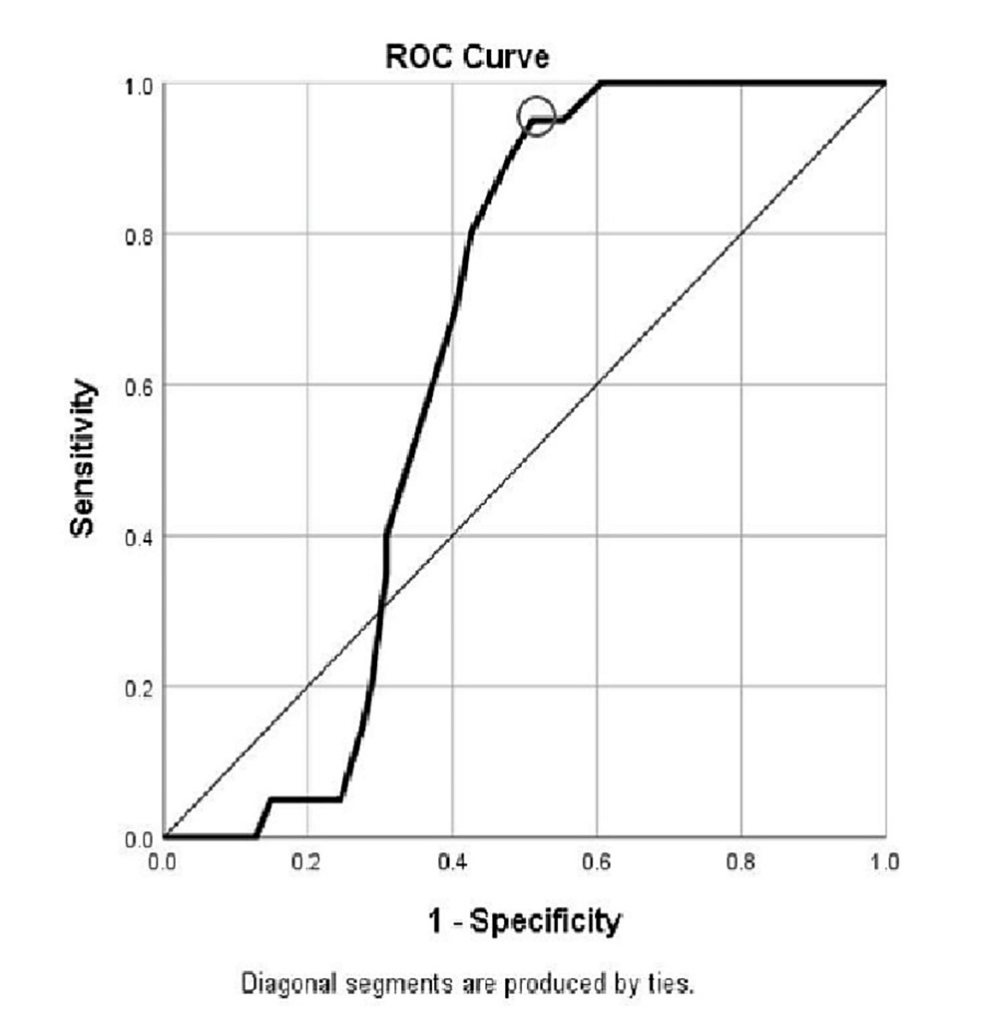
ROC curve to determine the cut-off of RRI ROC: receiver operating characteristic; RRI: renal resistive index

In order to improve renal survival, hemodialysis was started in a few of the patients with end-stage renal disease. Among them, the diabetic patients’ proportion was significantly higher compared to non-diabetic (p=0.003). Of the 28 patients who were started on hemodialysis, four belonged to stage IV and 24 to stage V of CKD. Hence, ROC analysis was performed to determine a cut-off of RRI values to distinguish stage IV and above stages of CKD from the lower stages. Using a cut-off of 0.655 for RRI, the area under the ROC curve to distinguish ≥IV stages of CKD from lower stages was 0.645 showing poor discrimination, with a sensitivity and specificity of 95% and 55%, respectively (Figure 2).

**Figure 3:**
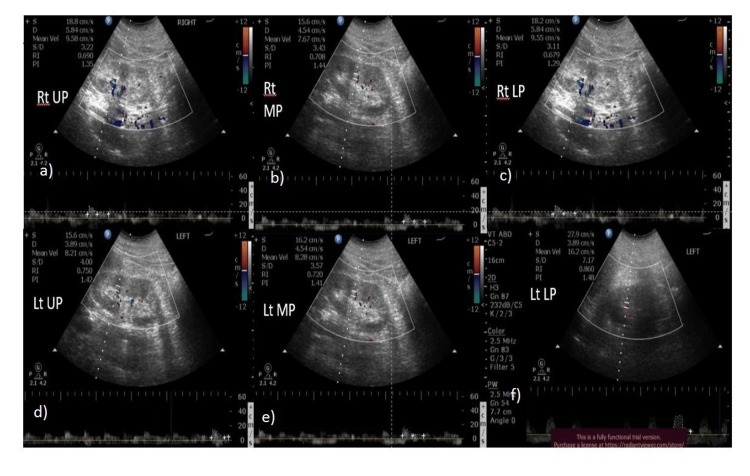
52-year-old diabetic male with stage IV CKD - average RRI = 0.73 RRI at each pole in the right kidney a) upper pole - 0.69, b) mid pole - 0.70, c) lower pole - 0.67. RRI at each pole in the left kidney d) upper pole - 0.75, e) mid pole - 0.72, f) lower pole - 0.86 RRI: renal resistive index

**Figure 4:**
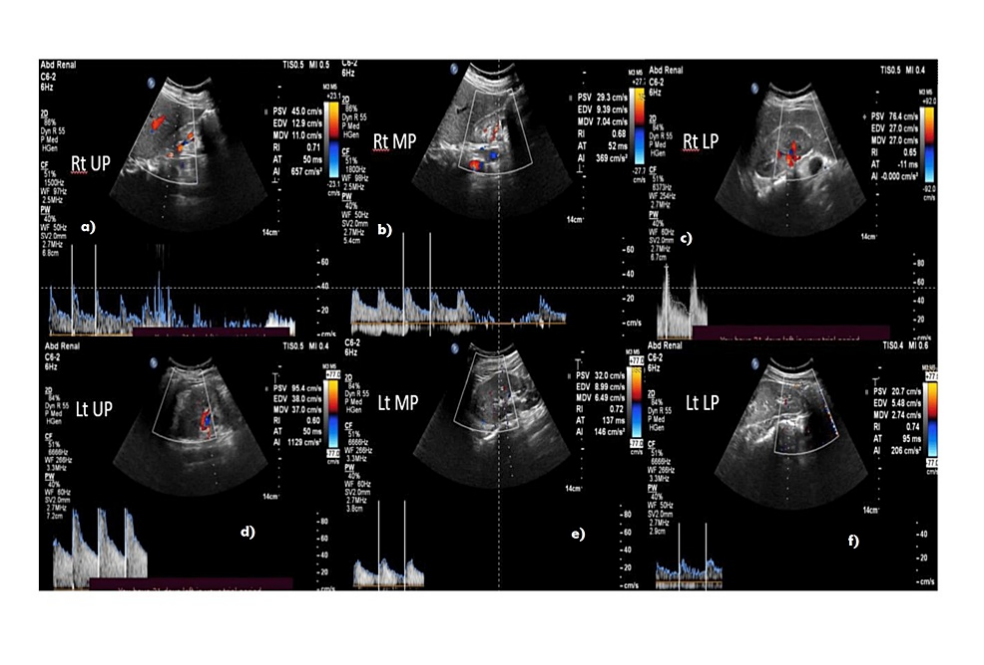
34-year-old non-diabetic female with stage IV CKD - average RRI = 0.68 RRI at each pole in the right kidney a) upper pole - 0.71, b) mid pole - 0.68, c) lower pole - 0.65. RRI at each pole in the left kidney d) upper pole - 0.60, e) mid pole - 0.72, f) lower pole - 0.74 RRI: renal resistive index

**Figure 5:**
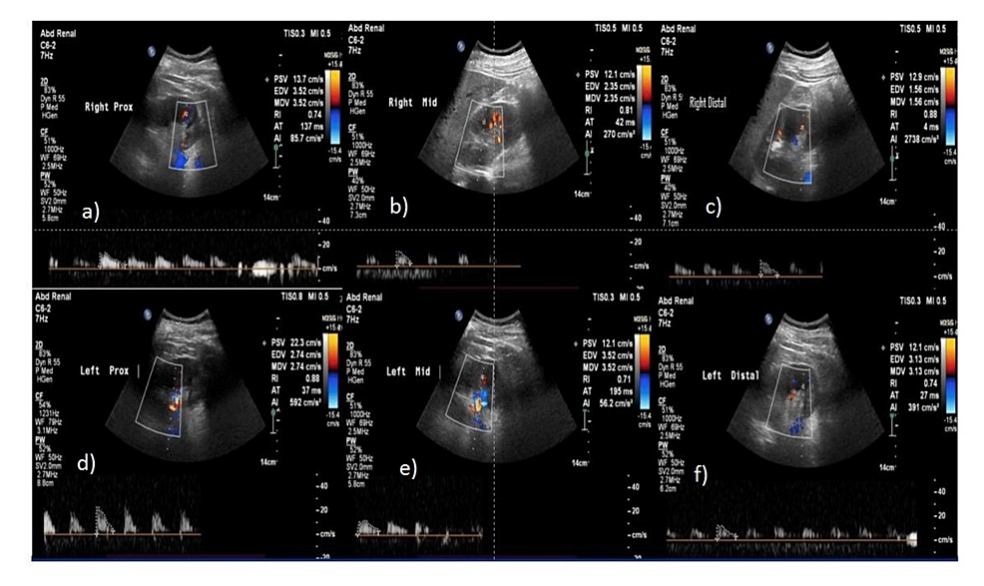
32-year-old diabetic female with stage V CKD - average RRI = 0.79 RRI at each pole in the right kidney a) upper pole - 0.74, b) mid pole - 0.81, c) lower pole - 0.88. RRI at each pole in the left kidney d) upper pole - 0.88, e) mid pole - 0.71, f) lower pole - 0.74 RRI: renal resistive index

**Figure 6:**
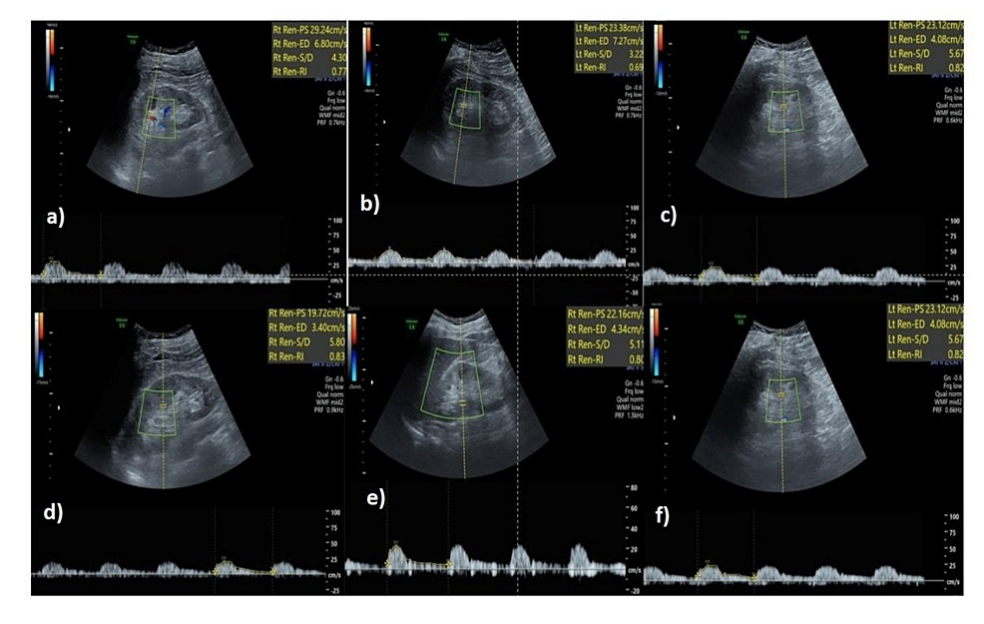
51-year old non-diabetic male with stage V CKD - average RRI = 0.78 RRI at each pole in the right kidney a) upper pole - 0.77, b) mid pole - 0.69, c) lower pole - 0.82. RRI at each pole in the left kidney d) upper pole - 0.83, e) mid pole - 0.80, f) lower pole - 0.82 RRI: renal resistive index

In our study, all the patients (51 in number) in ≥IV stages of CKD had a resistive index value of more than 0.655. However, we had 26 patients who were <IV stage of CKD and had a resistive index value of more than 0.655. The rest of the 37 patients of <IV stage of CKD had a resistive index less than 0.655 (Figures 3, 4, 5, 6).

## Discussion

Chronic kidney disease is universally considered a public health burden with increasing prevalence and incidence. It involves a range of pathophysiologic processes and is associated with several risk factors such as diabetes, hypertension, obesity, tobacco smoking, and dyslipidemia. Diabetes mellitus is the leading cause of CKD worldwide with diabetic nephropathy being a progressive kidney disease caused by microangiopathy of capillaries in the kidney glomeruli. There are various risk factors associated with Type 2 diabetes mellitus (DM) which include the patient’s age, duration of diabetes, systolic blood pressure, glycosylated haemoglobin (HbA1c), and renal volume. Girach et al performed a study and concluded that the strongest risk factors for DN are glycemic control and duration of diabetes. The modifiable risk factors were hypertension, hyperlipidemia, and smoking, and the unmodifiable risk factors were age at the onset of diabetes and genetic factors [7]. Another study conducted by Gall et al in 1997 reported that risk factors for the development of incipient or overt DN were increased baseline log urinary albumin excretion rate, male sex, presence of retinopathy, increased serum cholesterol concentration, glycosylated haemoglobin concentration and age [8].

eGFR has been in use for renal function screening since ages, however, it is difficult to assess the pathogenesis of CKD and predict the renal prognosis using only eGFR. Kawai T et al [9] in their study demonstrated that advanced CKD stages showed significantly higher RRI than patients with earlier stages of CKD. Also, it was concluded that subjects with diabetes showed a remarkably higher RRI than those without diabetes. Categorisation of patients into various stages of CKD showed a significant difference between RRI in diabetic and non-diabetic patients in stages I, II, and III. In our study, RRI showed a significant negative correlation with eGFR and was seen to progressively increase with rising stages of CKD. This could be explained by the primary glomerular involvement in the early stages of the disease, hence leading to a near-normal RRI [10]. Whereas in advanced stages, glomeruli turn sclerotic and tubules become atrophic with growing interstitial fibrosis. This is added by the advanced arteriosclerosis in intrarenal arteries at advanced CKD stages, contributing to an increase in RRI [11]. An interesting finding was that in stages I-IV of the disease, the absolute value of RRI in our study fell within the normal range (0.4-0.7). Given that baseline RRI varies among patients, a sequential increase is a better indicator of disease progression than an absolute figure that may be used as a cut-off (Table 1).

Additionally, in our study, RRI was significantly higher in diabetic patients compared to non-diabetics. So diabetes can accelerate vascular damage and RRI can detect this change more sensitively than eGFR. Mean RRI was significantly higher in diabetic patients in stages II and III of CKD, in comparison with those affected by other diseases, with an equivalent GFR. Whereas no significant difference was noted in stages I, IV and V. Greater proportion of patients in stage I of CKD in our study were non-diabetic (13 out of 19), as fewer stage-I diabetic patients were referred for renal biopsy. This may be the reason for no significant difference in stage I patients. Also, the influence of advanced loco-regional alterations (vascular and interstitial) on RRI exceeds systemic factors like pulse pressure and pulse wave velocity with decreasing GFR. Hence, this validates the need for additional incorporation of RRI in the routine protocol to assess the etiopathogenesis of CKD in the early stages. However, renal biopsy will remain the prime modality to assess the complete pathology and alterations of the diseased kidney.

Gopalakrishnan SS et al [12] had shown in their study that RRI correlated positively with blood urea nitrogen and serum creatinine. Moreover, Ishimura et al [13] came up with a positive relationship between RRI, creatinine clearance, and the age of diabetic patients. On the contrary, our study described that RRI independently correlated with serum creatinine with no significant association between RRI and the patient’s age. This accounts for the very little number of subjects aged over 60 years who were included in the study.

Kawai et al [9] had also shown a notable relationship between RRI and level of proteinuria, diastolic blood pressure, and pulse pressure, with no correlation between RRI and systolic blood pressure. Our study failed to provide a significant association between RRI and urine albumin excretion. This may be because not all patients were subjected to urine albumin testing. Also, in our institution, renal biopsy was attempted only in those diabetic patients who presented with nephrotic-range proteinuria and symptoms.

However, our study was consistent with the previous study on the relationship between RRI and systolic blood pressure. No significant correlation was demonstrated, which could be due to the increased number of patients who were subjected to medical treatment like anti-hypertensives at the time of examination. In the later stages of CKD, atherosclerotic arterial wall stiffening pursues, which reduces the elasticity and diastolic blood pressure with a rise in pulse pressure [14].

In this study, no relationship was established between renal volume and the presence of diabetes. Mancini et al [15] in their study had shown that the renal volume of diabetic patients was significantly higher than non-diabetic controls. In our study, we compared renal volume between diabetic CKD patients and subjects with other kidney diseases. Diabetic kidneys undergo hypertrophy with hyperperfusion in the early stages, followed by interstitial fibrosis and atrophy resulting in decreased renal size in advanced stages. In kidneys affected by other diseases, they don’t follow the hypertrophy-hyperperfusion mechanism. Hence, the renal volume could not define the etiopathogenesis of kidney disease.

Dialysis and renal transplant are effective means to treat end-stage nephropathy and help to replace the functions of kidneys when they no longer work. Diabetic nephropathy can cause a serious increase in the number of end-stage renal disease patients, requiring hemodialysis in their advanced stages. Chen Q et al [16] reported that diabetic nephropathy patients will suffer from serious kidney failure, with the probability of kidney failure much higher than that of normal kidney patients. Our study showed the commencement of hemodialysis to be more in diabetic patients compared to non-diabetic kidney disease patients, involving only stage IV and V cases.

An attempt was done to derive a cut-off value of RRI to distinguish stage IV and above stages of CKD from the lower stages. The results attained were highly sensitive with poor specificity (RRI cut off = 0.655), noting that it could be used as a screening tool. However, the relationship between RRI and the severity of CKD may not be highly robust, as the area under the ROC curve was <0.7. Hence, such a tool could be used to track renal damage progression, but with poor discrimination between the advanced stages (stage IV and V) from early CKD stages.

Limitations

Confounding factors such as age, gender, muscle mass, and BMI were not matched in the study. Most of the patients had already been under medical treatment for hypertension, diabetes, and dyslipidemia at the time of investigation, which would have affected various parameters. Baseline RRI values were not taken for the patients at the initial stages of CKD and a prospective continuous evaluation with serial RRI would have helped more in predicting the outcome.

## Conclusions

The renal resistive index could be considered a marker of renal function in both diabetic and non-diabetic kidney disease, with an ability to arrive at etiopathogenesis in the early stages. The resistive index correlated well with eGFR and serum creatinine indicating its role as a Doppler parameter, which can be used as complementary to biochemical parameters. With the increasing prevalence of diabetes, diabetic nephropathy remains the prime cause for the commencement of renal replacement therapy. Even though RRI could be used as a screening tool for kidney disease, it does not provide a definite cut-off for efficient discrimination between early and advanced CKD stages. As there is individual diversity with regard to baseline RRI, a sequential increase in RRI is a better sign of disease progression than an absolute value.
